# Anatomy and white matter connections of the fusiform gyrus

**DOI:** 10.1038/s41598-020-70410-6

**Published:** 2020-08-10

**Authors:** Ali H. Palejwala, Kyle P. O’Connor, Camille K. Milton, Chris Anderson, Panayiotis Pelargos, Robert G. Briggs, Andrew K. Conner, Daniel L. O’Donoghue, Chad A. Glenn, Michael E. Sughrue

**Affiliations:** 1 Department of Neurosurgery, University of Oklahoma Health Sciences Center, Oklahoma City, OK USA; 2 Department of Cell Biology, University of Oklahoma Health Sciences Center, Oklahoma City, OK USA; 3 Department of Neurosurgery, Prince of Wales Private Hospital, Sydney, NSW 2031 Australia

**Keywords:** Anatomy, Nervous system

## Abstract

The fusiform gyrus is understood to be involved in the processing of high-order visual information, particularly related to faces, bodies, and stimuli characterized by high spatial frequencies. A detailed understanding of the exact location and nature of associated white-tracts could significantly improve post-operative morbidity related to declining capacity. Through generalized q-sampling imaging (GQI) validated by gross dissection as a direct anatomical method of identifying white matter tracts, we have characterized these connections based on relationships to other well-known structures. We created the white matter tracts using GQI and confirmed the tracts using gross dissection. These dissections demonstrated connections to the occipital lobe from the fusiform gyrus along with longer association fibers that course through this gyrus. The fusiform gyrus is an important region implicated in such tasks as the visual processing of human faces and bodies, as well as the perception of stimuli with high spatial frequencies. Post-surgical outcomes related to this region may be better understood in the context of the fiber-bundle anatomy highlighted by this study.

## Introduction

The fusiform gyrus, also known as the occipitotemporal gyrus, is a structure spanning the basal surface of the temporal and occipital lobes^1^. It is the largest component of the human ventral temporal cortex, a functionally-defined region critical for visual categorization^1,2^. Although the precise function of the fusiform gyrus has not yet been entirely revealed, it has been implicated in high-level tasks regarding visual processing, particularly the processing of information related to faces^3–5^, bodies^6,7^, and stimuli characterized by high spatial frequencies^8^.

The connection between the anatomical structure of the fusiform gyrus and this region’s function remains contested^2,7^. Clarification of the fiber-bundle anatomy and functional relationships inherent to the fusiform gyrus may have relevance to clinical applications such as operative planning for glioma resection and awake craniotomies. In addition, human neuroimaging studies have found that certain regions of the fusiform gyrus and its underlying white matter connections are related to a variety of conditions, such as developmental prosopagnosia^5,9,10^, pure alexia^8,11^, and anorexia nervosa. Nevertheless, direct correlations between surgical anatomy and functional connectivity of the fusiform gyrus have not been previously described in neurosurgical literature.

A white matter tract identification technique consisting of gross brain dissection and generalized q-sampling tractography has been implemented previously to suggest links between form and function for other brain regions^12,13^. Here we investigate the structural organization and network connectivity of the fusiform gyrus. Through GQI-based fiber tracking validated by gross dissection as a direct anatomical method of determining white matter connections^14^, we have characterized these tracts based on key connections and anatomical relationships to other structures.

## Materials and methods

### Definition of the region of interest

**Figure 1:**
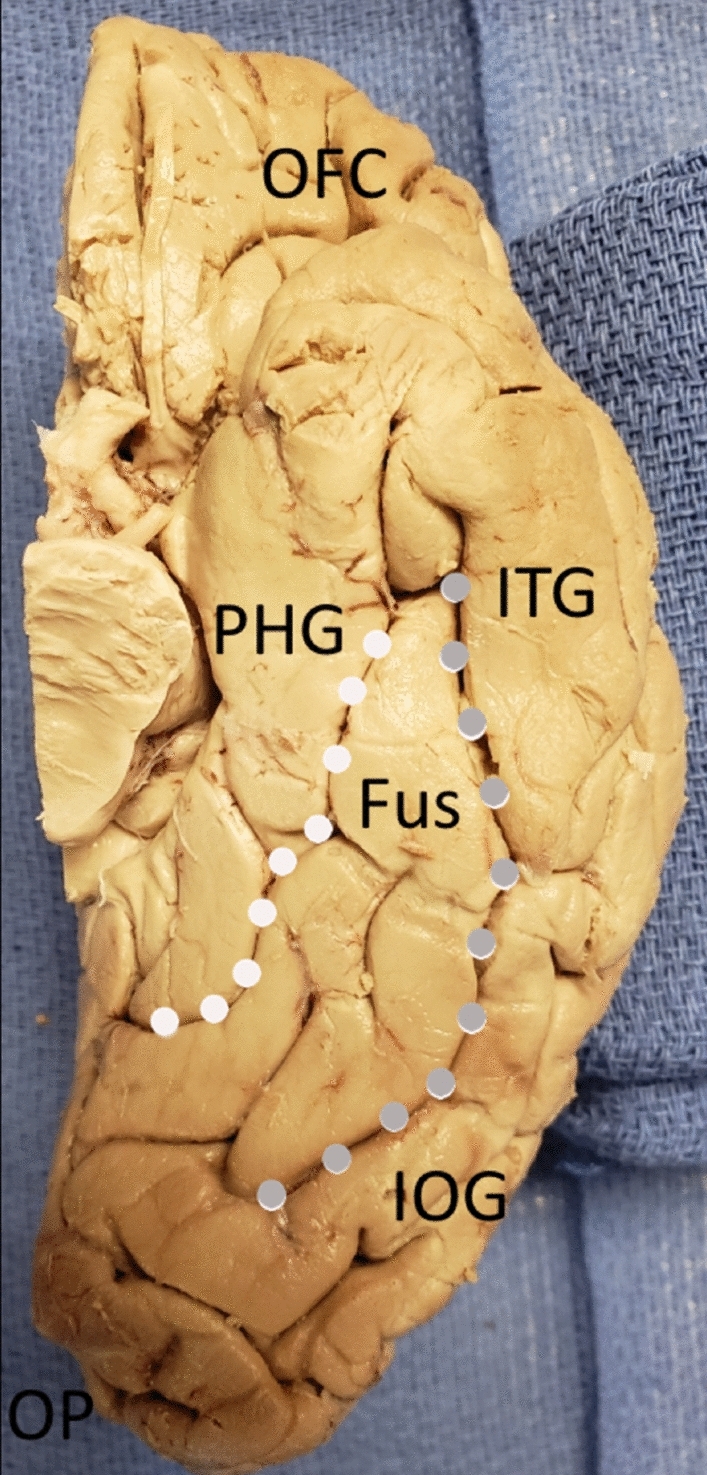
The boundaries of the fusiform gyrus. The fusiform gyrus viewed from the basal surface of the right cerebral hemisphere. It is bounded medially by the collateral sulcus (white dots). It is bounded laterally by the occipito-temporal sulcus (grey dots). It extends anteriorly towards the temporal pole. CS (collateral sulcus)—white dots, PHG = parahippocampal gyrus, Fus = fusiform gyrus, OTS (occipitotemporal sulcus) = grey dots, IOG = inferior occipital gyrus, ITG = inferior temporal gyrus, OP = occipital pole, OFC = orbito-frontal cortex.

The fusiform gyrus is located on the basal surface of the occipital and temporal lobe. The fusiform gyrus is bounded medially by the collateral sulcus, which separates it from the parahippocampal gyrus. It is bounded by the occipito-temporal sulcus laterally, which separates it from the inferior occipital gyrus and inferior temporal gyrus anteriorly. The anterior border of the fusiform gyrus was noted to be towards the temporal pole. The fusiform gyrus does not extend to the occipital pole; therefore, we cannot view the gyrus from the dorsal surface of the occipital lobe from a posterior view. This can be seen in Fig. 1.

### GQI tractography

Imaging data from the Human Connectome Project (HCP) was obtained for this study from the HCP database (https://humanconnectome.org, release Q3). Diffusion imaging from ten healthy adult controls was analyzed: 100,307, 103,414, 105,115, 110,411, 111,312, 113,619, 115,320, 117,122, 118,730, and 118,932. MRI specifications include: 3 T (3 T), 32-channel head coil, TR of 0.4 s, TE of 33 ms, FoV 208 mm in the read direction (anterior–posterior), 180 mm in the phase encoding direction, and 144 mm in the inferior–superior direction. A multishell diffusion scheme was used, and the b-values were 990, 1985, and 1980s/mm^2^. Each b-value was sampled in 90 directions. The in-plane resolution was 1.25 mm. The slice thickness was 1.25 mm. The diffusion data were reconstructed using generalized q-sampling imaging with a diffusion sampling length ratio of 1.25^15^.

We have utilized similar procedures for GQI tractography in prior studies^12,13^. We performed brain registration to Montreal Neurologic Institute (MNI) space, wherein imaging is warped to fit a standardized brain model for comparison between subjects^16,17^. Tractography was performed in DSI Studio (University of Pittsburgh) using two predefined regions of interest (ROIs) to isolate single tracts^15,18^. ROIs were traced to delineate the fusiform gyrus in the left and the right side of the cerebrum. Voxels within each ROI were automatically traced with randomized seeding of the voxel and/or tract with a maximum angular threshold of 45°. When a voxel was approached with no tract direction or a direction change greater than 45°, the tract was halted. Tractography was stopped after reaching a length of 450 mm. In some instances, exclusion ROIs were placed to exclude obviously spurious tracts that were not involved in the network of interest. All tracts were dissected in both hemispheres for all regions of the fusiform gyrus. Laterality was defined as volume differences between contralateral identical tracts. Volumes were generating using the tractography software. A two-tailed paired sample T-test (T-test for dependent means) was performed in order to compare mean white matter tract volumes for the right and left hemispheres.

**Figure 2:**
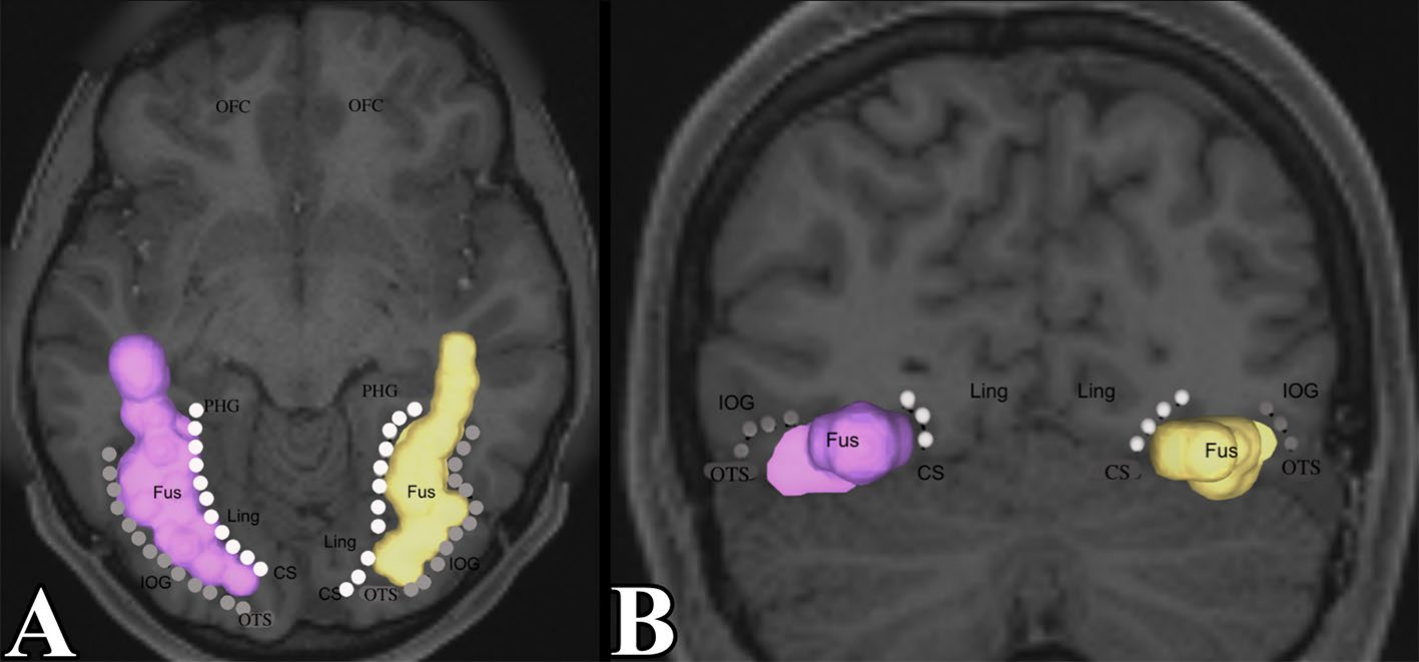
The fusiform gyrus region of interest used in tractography. (**A**) Demonstrates an axial section of the fusiform gyrus. The collateral sulcus and occipito-temporal sulcus were identified to bound the fusiform gyrus from a medial–lateral direction. It was extended towards the temporal pole. We see that the fusiform gyrus does not reach the posterior-most aspect of the occipital lobe. (**B**) Demonstrates a coronal section of the fusiform gyrus. CS (collateral sulcus) = white dots, PHG = parahippocampal gyrus, Fus = fusiform gyrus, OTS (occipitotemporal sulcus) = grey dots, IOG = inferior occipital gyrus, ITG = inferior temporal gyrus, OP = occipital pole, OFC = orbito-frontal cortex.

To generate tracts, ROIs was placed manually into the region of interest defined as the fusiform gyrus. Figure 2 demonstrates the fusiform gyrus bounded by the collateral sulcus medially and occipito-temporal sulcus laterally. Anteriorly, it extends towards the temporal pole. ROIs were placed in the other gyri of the brain. Doing this allowed us to investigate all other white matter tracts between the fusiform gyrus and the rest of the cerebral cortex.

### Postmortem dissection

Informed consent was obtained from legally authorized representatives or next of kin prior to cadaver dissection. All methods were carried out in accordance with local ethical guidelines and regulations for the use of human cadaver specimens. All experimental protocols were approved by the University of Oklahoma Health Sciences Center Willed Body Program. The purpose of the post-mortem dissections was to demonstrate the location of major tracts connecting to the lateral occipital lobe. Postmortem dissections were performed using a modified Klingler technique^19^. While there have been documented modifications to this technique^19,20^, it was first described by Ludwig and Klingler et al. in 1956^21^. Initial requirement was to freeze the brains to at least − 10°C^21^. An example of brains frozen to temperatures up to − 80 °C is demonstrated by David et al.^20^. The most comprehensive analysis of this technique is described by Wysiadecki et al.^19^. There is no documented advantage to freezing temperatures beyond this point. This technique can also be used when tractography data does not match the dissection specimens^12^. Refreezing of specimens is recommended if subsequent dissections are undergone after an extended period of time (1-month) after previous dissections were completed^22^. Ten specimens were used for this study, obtained from our institution’s Willed Body Program with approval of the state’s anatomical board. The cadaveric brains were fixed in 10% formalin for at least 3 months after removal from the cranium. Up until the time of dissection, the pia-arachnoid membrane was left attached.

After fixation with formalin, specimens were rinsed with water for two days, and then frozen at − 10 °C for 8 h. Rinsing brains reduced formaldehyde exposure. After thawing, dissection of the specimens began with removal of the meninges and identification of cortical anatomy, including gyri and sulci. The brains were frozen to ease white matter dissections. The water molecules between the myelinated nerve fibers which partially separates them and allows easier dissection of bundles or tracts of fibers. The fibers are still connected and contiguous between their origin and termination so they are not disrupted. Relevant cortical areas were identified first. Starting superficially, they were then peeled back to reveal white-matter areas of interest. Care was taken to leave cortical areas corresponding to white-tracts of interest intact in order to preserve their relationship. Tracts were dissected with blunt instruments to avoid disrupting the natural tract anatomy. Photographs were taken at each stage of the dissection. To aid dissection, surgical loupes and adequate lighting were used. This allowed for greater visualization during micro-dissections.

For dissections, hemispheres of brains were placed on flat surfaces. The tractography data was present within view for proper tract localization. We started by finding the endpoint of a tract whether it was the origin or insertion and dissecting out the cortical terminations. Next we followed the tract to the other endpoint by carefully removing the superficial grey and white matter just adjacent to the already exposed white matter tract and continuing to dissect until the other endpoint was found. For example, when we dissected the connection between the lingual gyrus and the fusiform, we first found the cortical termination within the lingual gyrus then carefully dissected anteriorly by removing superficial brain parenchyma in a posterior to anterior direction until we found the cortical termination within the fusiform gyrus. This was repeated for all specimens.

## Results

### Local connections with the lingual gyrus

**Figure 3:**
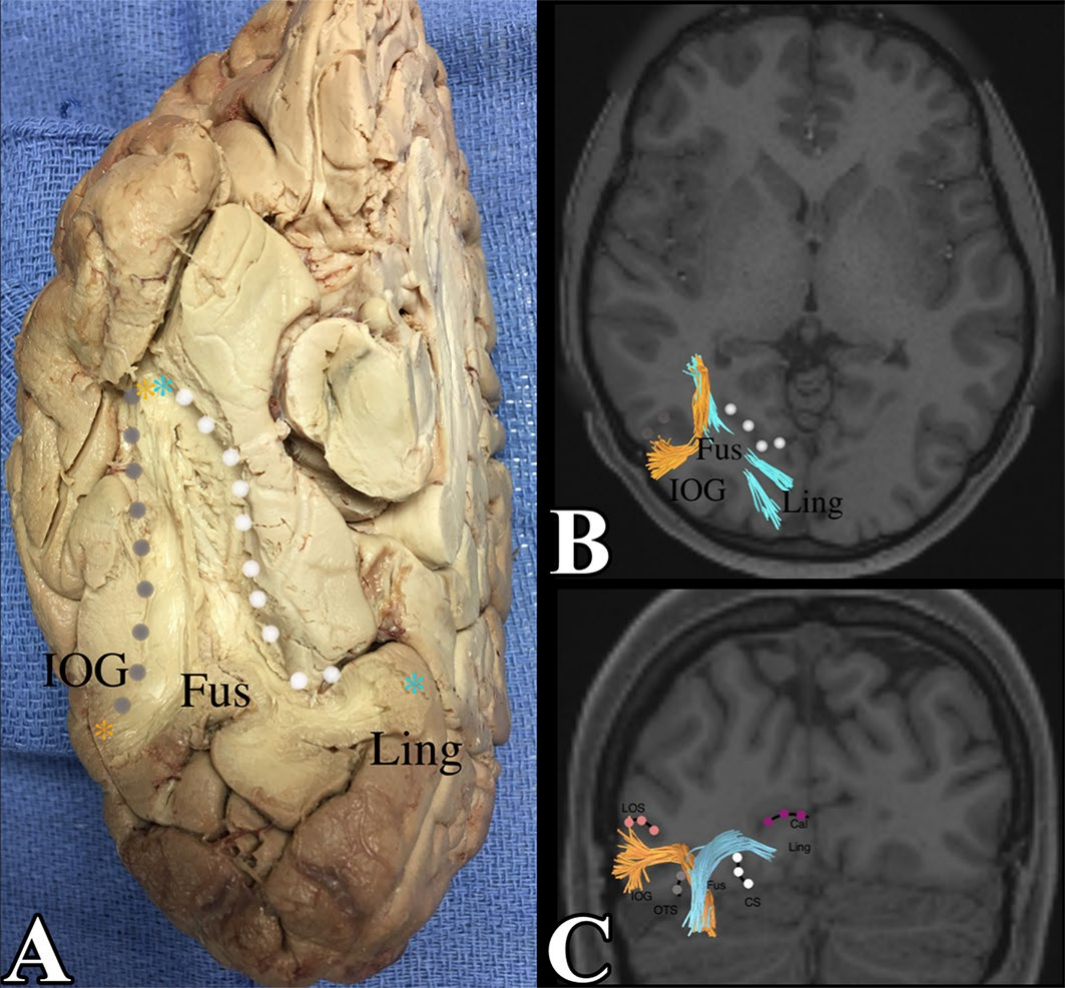
(**A**) Demonstrates a basal view of the right side of the fusiform gyrus connections with the inferior occipital gyrus and the lingual gyrus. The collateral sulcus, bounding the gyrus medially, and the occipito-temporal sulcus were dissected to approach the white matter core of the fusiform gyrus. Note how fibers have one ending in the medial portion of the lingual gyrus, course over the collateral sulcus and another termination in the anterolateral fusiform gyrus. In a similar manner, there are fibers with an ending in the lateral portion of the inferior occipital gyrus that course over the occipito-temporal sulcus, with another termination in the anterolateral fusiform gyrus. (**B**) Demonstrates an axial section of the occipital lobe, when viewed from above, with these right sided fibers between the lingual gyrus/inferior occipital gyrus and the anterolateral fusiform gyrus. (**C**) A coronal section when viewed from the front, demonstrates these fibers heading away from the page, anteriorly. IOG = inferior occipital gyrus, OTS (occipito temporal sulcus) = grey dots, Fus = fusiform gyrus, CS (collateral sulcus) = white dots, Ling = lingual gyrus, Cal (calcarine sulcus) = purple dots, LOS (lateral occipital sulcus) = pink dots.

**Figure 4:**
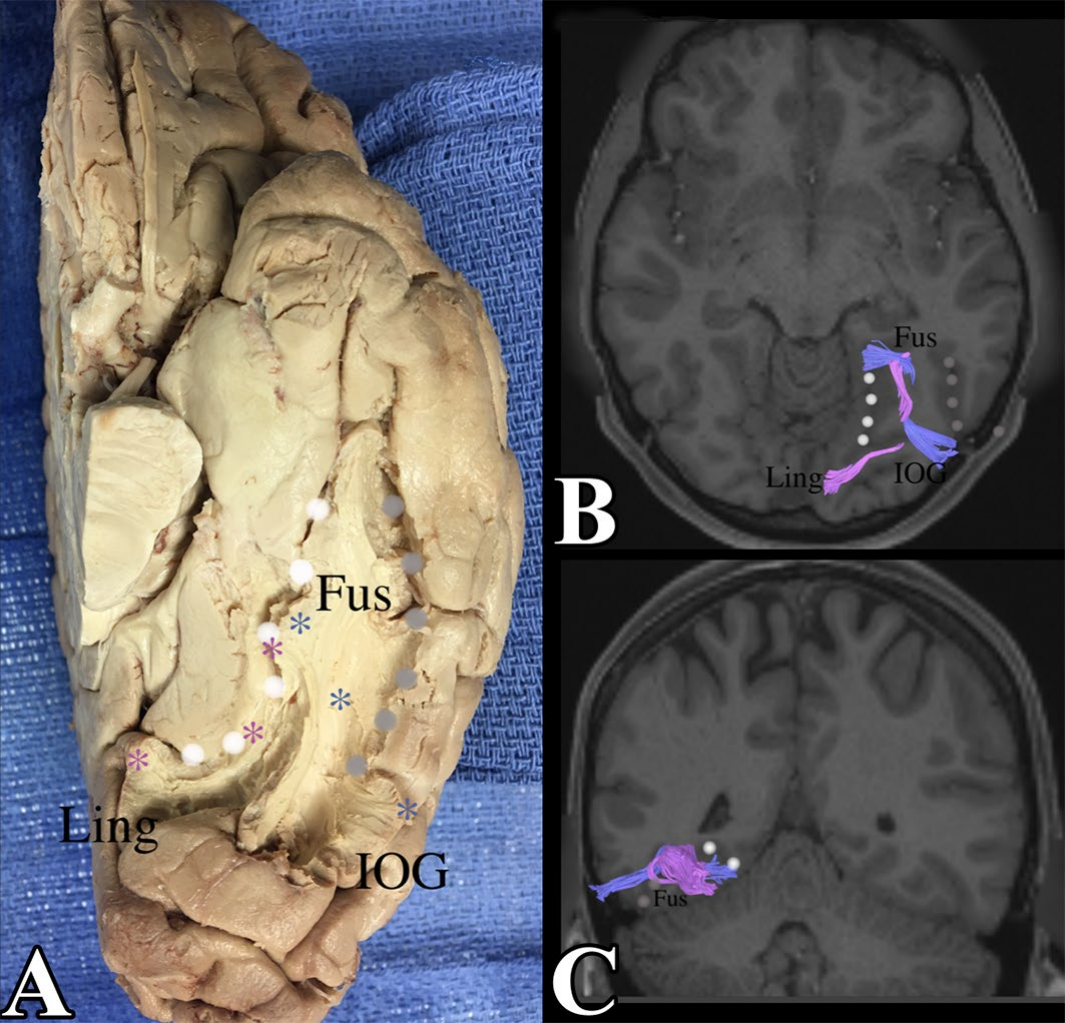
Left sided fusiform gyrus connections with the inferior occipital gyrus and the lingual gyrus. (**A**) Demonstrates a dissection with the grey matter between the collateral sulcus and the occipito-temporal sulcus cored out. It confirms the presence of fibers from the inferior occipital gyrus and lingual gyrus feeding into the fusiform gyrus. (**B**) And axial section viewed from below, demonstrates the fibers from the left inferior occipital gyrus and lingual gyrus feeding into the fusiform gyrus. (**C**) A coronal section of the brain when viewed from behind, demonstrates the fibers heading into the page (anteriorly), towards the antero-medial fusiform gyrus, adjacent to the collateral sulcus. IOG = inferior occipital gyrus, OTS = grey dots, Fus = fusiform gyrus, CS = white dots, Ling = lingual gyrus.

The fusiform gyrus receives inputs from the lingual gyrus. One location of fiber termination was identified in the posteromedial portion of the lingual gyrus. These fibers course over the collateral sulcus and enter the fusiform gyrus, terminating in the anterior portion of the fusiform gyrus. On the left side, we noted that these fibers, in a serpiginous manner, coursed over the collateral sulcus, ending in the anteromedial portion of the fusiform gyrus. On the right side, we noted that similar fibers also coursed over the collateral sulcus, with the location of the fusiform termination in the anterolateral portion of the fusiform gyrus. We confirmed this through dissection. The collateral sulcus was identified and the grey matter between it was cored out to expose the white matter between the lingual gyrus and the fusiform gyrus. It confirmed the presence of the fibers identified by tractography. The local connections between the fusiform and lingual gyrus are demonstrated in Figs. 3 and 4.

U-shaped fibers were also detected between the lingual gyrus and the fusiform gyrus. These adjacent gyri, which are separated by the collateral sulcus, had a U-shaped fiber that coursed over the collateral sulcus to connect the two gyri at the superficial dorsal surface of the occipital lobe.

### Local connections with the inferior occipital lobe

The fusiform gyrus also has connections with the inferior occipital lobe. These connections primarily originate on one side from the posterolateral portion of the inferior occipital gyrus and loops up over the occipital–temporal sulcus. These fibers also ended up in the anterior most portion of the fusiform gyrus in an analogous manner to the fibers from the lingual gyrus (mentioned previously). In the same manner, we observed that the fibers ended in the anteromedial portion of the fusiform gyrus on the left and the anterolateral portion of the gyrus on the right. These fibers were confirmed in dissection. The occipito-temporal sulcus was identified and the grey matter on each side was cored out to expose the white matter between inferior occipital gyrus and the fusiform gyrus. It confirmed the presence of fibers between the inferior occipital gyrus and the fusiform gyrus as described. The connections between the right and left fusiform gyrus with the inferior occipital gyrus and the lingual gyrus can be seen in Figs. 3 and 4.

U-shaped fibers were also detected between the inferior occipital gyrus and the fusiform gyrus. These adjacent gyri, which are separated by occipito-temporal sulcus, had a U-shaped fiber that coursed over the sulcus to connect the two gyri at the dorsal surface of the occipital lobe.

### Vertical occipital fasciculus (VOF)

**Figure 5:**
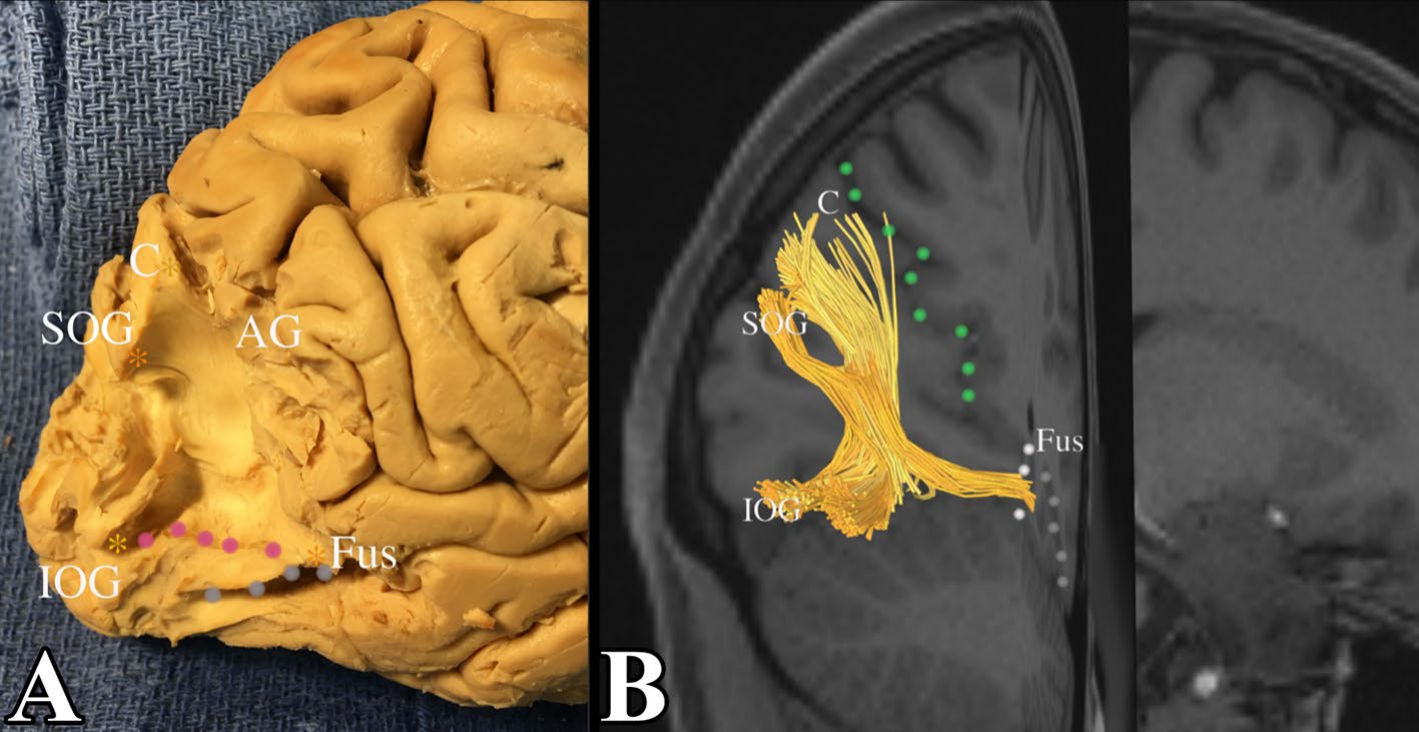
Vertical occipital fasciculus. (**A**) Demonstrates a dissection deep into the white matter core of the occipital lobe. We first exposed connections between the superior occipital gyrus and the inferior occipital gyrus. Note how the fibers course obliquely from the superior occipital gyrus to the inferolateral occipital cortex (pink dots form the superior aspect the inferior occipital gyrus). The gross dissection represents the lateral portion of the VOF. We subsequently followed this back to expose a subcomponent of the VOF between the cuneus and fusiform gyrus. This is confirmed in tractography in (**B**). POS (parieto-occipital sulcus) = green dots, C = cuneus, CS (collateral sulcus) = white dots, Fus = fusiform gyrus, SOG = superior occipital gyrus, IOG = inferior occipital gyrus, OTS – grey dots, LOS (lateral occipital sulcus) = pink dots.

A fiber tract was also noted between the fusiform gyrus and the cuneus. These fibers had one ending located at the posteromedial portion of the cuneus. These fibers coursed downwards and then bended laterally, past the collateral sulcus, to reach the anteromedial portion of the fusiform gyrus in both left and right side. These were fibers that were part of the VOF. We confirmed this by initiating dissection at the superior occipital gyrus. The white matter was exposed and followed to inferior occipital gyrus to reveal the lateral most portion of the VOF. From there, we were able to extend the dissection medially to the cuneus, which is bound anteriorly by the parieto-occipital sulcus. From there, we extended the dissection anteriorly and inferiorly to find the terminations in the fusiform gyrus. The position in the white matter of the fusiform gyrus was confirmed by locating the occipito-temporal sulcus laterally and the collateral sulcus medially. An example of this can be seen in Fig. 5. Included are portions of the VOF that involve the lateral occipital lobe, with terminations in the superior occipital lobe and inferior occipital lobe.

### Long range association fibers

**Figure 6:**
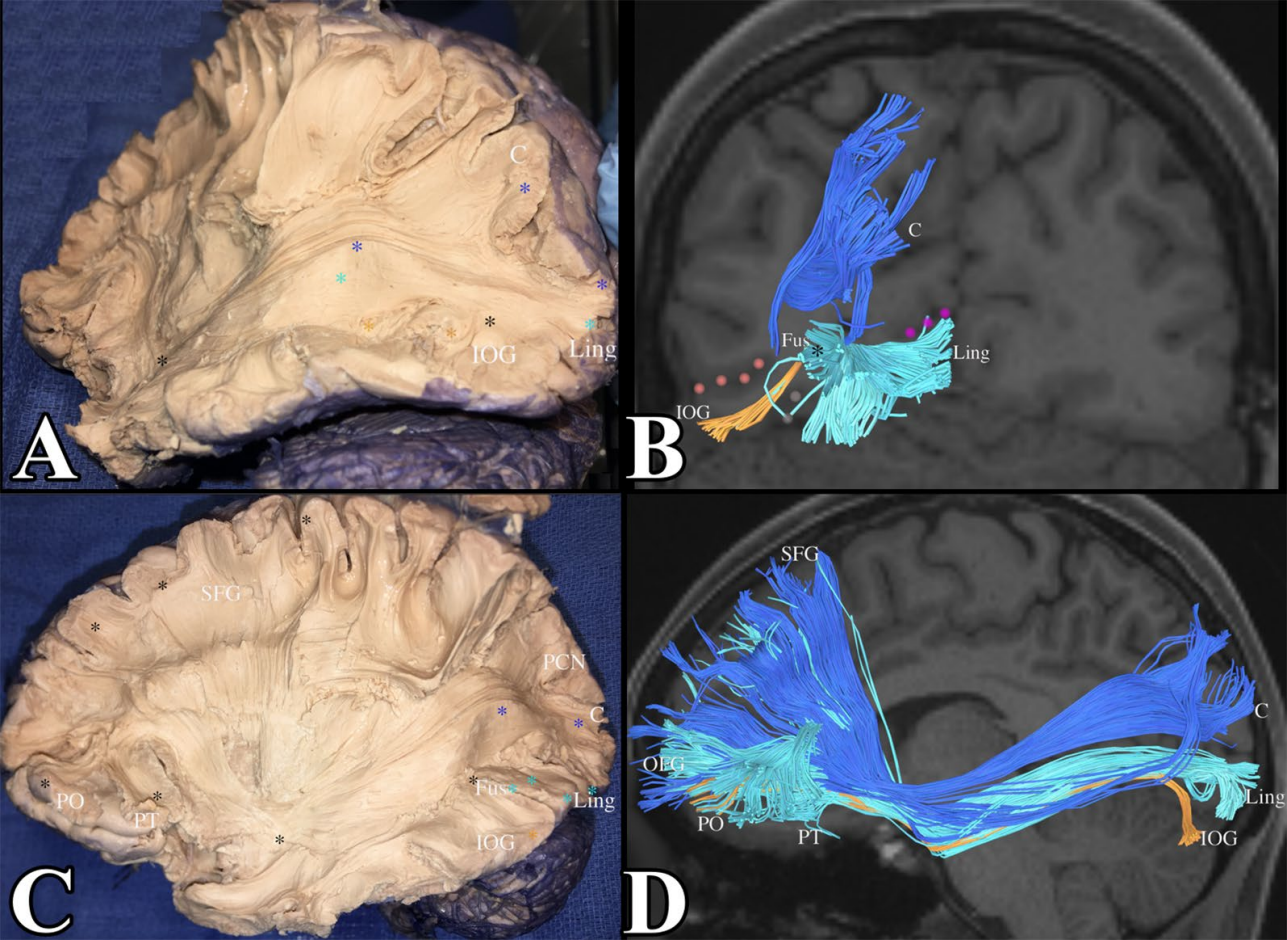
(**A**) Demonstrates a dissection of the IFOF. Notice its subcomponents from the cuneus (demarcated in blue), lingual gyrus (light blue), and the inferior occipital gyrus (orange). This is confirmed in tractography in (**B**). The lateral occipital sulcus was included in pink dots to localize the fibers in the inferior occipital gyrus. The calcarine sulcus was included in purple dots to localize the lingual gyrus. We can see that subcomponents of the IFOF from the lingual gyrus and inferior occipital gyrus meet in the fusiform gyrus (black star) and course through the fusiform gyrus to reach the temporal lobe and their final destination in the frontal lobe. (**C**,**D**) Demonstrate the full IFOF. C = cuneus, L = lingual gyrus, IOG = inferior occipital gyrus, LOS (lateral occipital sulcus) = pink dots, CS (collateral sulcus) = purple dots.

**Figure 7:**
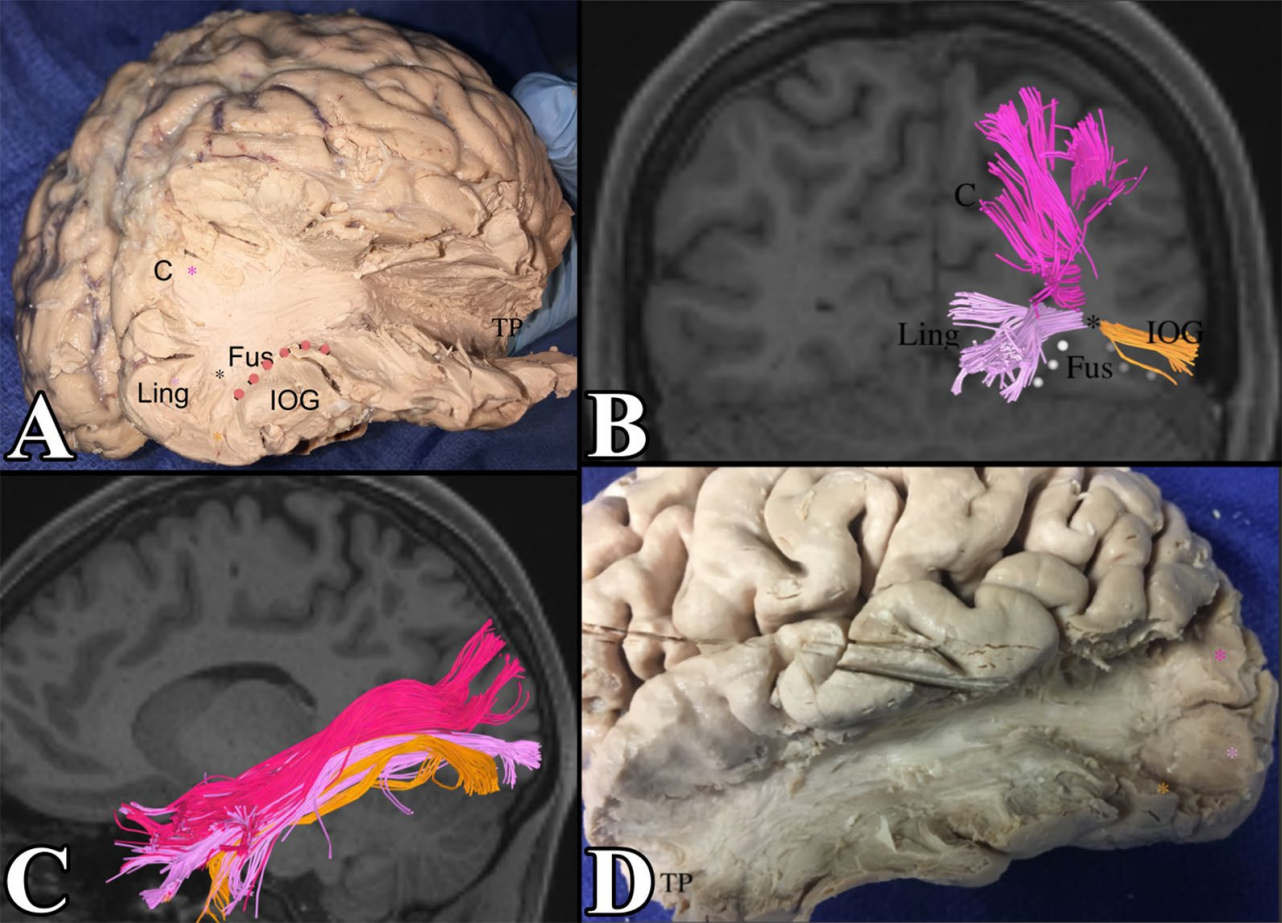
(**A**) Demonstrates a dissection of the ILF. Notice its subcomponents from the cuneus (demarcated in pink), lingual gyrus (light purple), and the inferior occipital gyrus (orange). The lateral occipital sulcus was included in pink dots to localize the fibers in the inferior occipital gyrus. This is confirmed in tractography in (**B**). We can see that subcomponents of the ILF from the lingual gyrus and inferior occipital gyrus meet in the fusiform gyrus (black star) and course through the fusiform gyrus to reach the temporal lobe. (**C**,**D**) Demonstrate the full ILF. C = cuneus, L = lingual gyrus, IOG = inferior occipital gyrus, LOS (lateral occipital sulcus) = pink dots.

The fusiform gyrus also serves as a conduit for the major association fibers of the occipital lobe. The inferior longitudinal fasciculus (ILF) and the inferior frontal occipital fasciculus (IFOF) have portions originating in the lingual gyrus and the inferior occipital gyrus that meet in the fusiform gyrus, course through the fusiform gyrus, before reaching their terminations in the temporal pole and frontal lobe respectively. We show the IFOF in Fig. 6 and the ILF in Fig. 7 to remind the reader of the association that the IFOF and ILF have with the white matter of the fusiform gyrus. Though we were not able to identify subcomponents of the ILF and IFOF that originated within the fusiform gyrus, we found that these subcomponents constituted a portion of the white matter of the gyrus.

**Figure 8:**
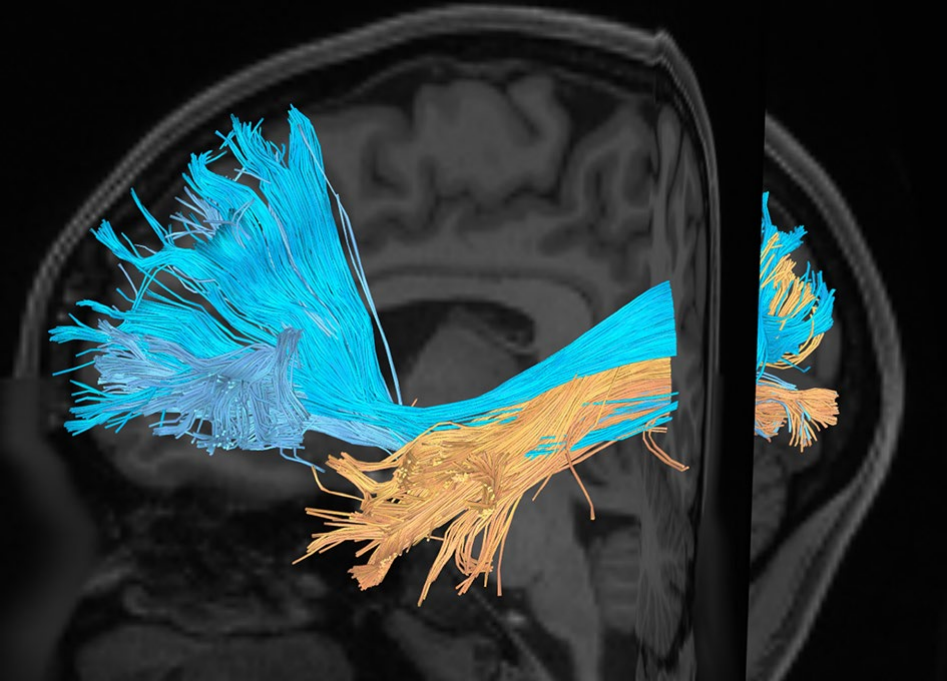
The IFOF and ILF demonstrated. The fibers are intermingling in the occipital lobe. Once reaching the level of the inferior parietal lobule, at the atrium of the lateral ventricle, the ILF diverges laterally to the temporal pole; the IFOF remains medially to course towards the frontal lobe.

The IFOF and ILF lie in close association with one another. The ILF is slightly ventral and superficial in relation to the IFOF. Once past the atrium of the lateral ventricle, the ILF proceeds more laterally to terminate in the temporal pole as shown in Fig. 8.

## Discussion

In this study, we addressed the underlying subcortical white matter anatomy of the fusiform gyrus, a temporal lobe region that is thought to play an important role in specialized visual processing functions, such as facial processing. To our knowledge, no previous study has described the subcortical anatomy of the fusiform gyrus and its relevance to neurosurgery. Sparing of function in cerebral surgery is dependent on the preservation of the white matter tracts linking functional areas. Here we have demonstrated the connections of the fusiform gyrus using diffusion spectrum imaging and confirmed these connections through gross anatomical dissection.

The roles of micro-dissection are well described in a recent publication^23^. The primary importance of micro-dissection is confirmation of tractography data since tractography is only a computer-generated representation of white matter tracts. The importance of identifying these tracts are to discuss the anatomical relationships with neighboring pathways. An example within neurosurgery would be masses that affect one tract would have higher likelihood of affecting neighboring tracts. Also, surgeons must understand what superficial tracts must be preserved when accessing deeper portions of the brain. Different techniques are emerging to aid in micro-dissection such as the photogrammetric imaging analysis^24^. This method works by taking imaging data of the specimen to reconstruct the cortical surface and between each step in dissection to minimize white matter loss during dissection.

For our dissection, we chose to follow the Klingler method as described previously. The Klingler method of fiber dissection has inherent strengths and weakness. The primary strength involves improved fiber resolution at final dissection through improved fiber separation by penetrating water molecules that expand the spaces between fibers during freezing. This allows ease of dissection as the fibers are already separated from adjacent fibers and brain matter before attempting dissection. The weakness of this dissection and dissections in general is that superficial fibers must be sacrificed when dissecting deeper fibers. Furthermore, for this technique, there must be a method for freezing brains to − 80° which may be unavailable to some researchers.

### Summary of results section

The first main bundle was the connection between the fusiform gyrus and the lingual gyrus. While the contralateral tracts originated within the same areas of the bilateral lingual gyri, there were slightly different insertion points on the fusiform gyrus (on the left the insertion was on the anteromedial portion of the gyrus and on the right it was on the anterolateral portion). There were also connections between the inferior occipital lobes (origin) and fusiform (insertion) bilaterally. The insertion points into the fusiform gyri were identical to the ones identified for the tracts between the lingual and fusiform gyri. Also U-fibers connected adjacent gyri between the inferior occipital and fusiform gyri. There was also a tract between the fusiform gyrus and the cuneus that was a part of the vertical occipital fasciculus. The vertical occipital fasciculus traditionally connects the superior and inferior occipital gyri. The portion inserting into the fusiform attached to the anteromedial portion of that gyrus. All of these connection demonstrate a strong connection between the occipital lobe and the fusiform gyri which suggests that the fusiform is involved in functions related to vision or processing of visual information.

### Fusiform gyrus and facial perception

Studies on the functionality of the fusiform gyrus support a link between this region and high-order visual recognition processes, particularly the recognition of faces^3–5,25^. The fusiform face area (FFA), within the right mid-fusiform gyrus has been particularly associated with facial processing^9^. Laterality of facial-processing function was determined in a study combining electrocorticography with electrical brain stimulation study performed by Rangarajan et al.^4^. In this study, the authors found that stimulation of the right fusiform gyrus could produce perceptual distortions when viewing faces. Notably, stimulation of the left fusiform led to unrelated visual distortions, including distortion of colors or the production of phonemes.

Using tractography and dissections, we demonstrated that the lingual gyrus and inferior occipital gyri feed into multiple areas of the anterior fusiform gyrus. On the left side, these connections were located in the medial fusiform gyrus, in an area known as the ventromedial visual area (VMV). VMV may play a role in integrating color, form, and texture information for holistic recognition^5,26^. Connections on the right side terminated in the anterolateral portion of the fusiform gyrus, in the previously mentioned fusiform face area, an area that is critical for processing facial features^27^.

Our connectivity studies support the fact that the fusiform gyrus may have different functions depending on laterality. In our dissection of 10 cadaveric human brains, we found that 7 brains demonstrated this consistent difference in fusiform terminal connections dependent on laterality. In one brain, we were not able to determine the terminal connection on both sides. In 2 other brains, we were unable to determine the terminal connection on one side. We were unable to determine the location of some terminal connections of local connections secondary to inability to find the fiber tract or preserve the tract in dissection. White matter tract volumes were not found to differ significantly between the right and left hemispheres (t = 0.56, *p* = 0.59). Table 1 displays the data for all anatomically-determined terminal connections of the fusiform gyrus.

**Table 1:** Location of local connection terminations in the fusiform gyrus.

Subject	Laterality	Location of local connection termination
1	R	Anterolateral
	L	Anteromedial
2	R	Anterolateral
	L	Anteromedial
3	R	Anterolateral
	L	Anteromedial
4	R	Undetermined
	L	Undetermined
5	R	Anterolateral
	L	Undetermined
6	R	Anterolateral
	L	Anteromedial
7	R	Undetermined
	L	Anteromedial
8	R	Anterolateral
	L	Anteromedial
9	R	Anterolateral
	L	Anteromedial
10	R	Anterolateral
	L	Anteromedial

*R* right, *L* left.

Aside from laterality, the perception of faces is thought to be mediated by a highly interconnected neural network, thought to include a core set of posterior regions including the occipital face area (OFA), the fusiform face area (FFA), the posterior superior temporal sulcus (pSTS), as well as so-called extended regions that include the anterior temporal pole, the amygdala, and the ventromedial prefrontal cortex^5^. Lohse et al.^10^ put forth a network model in which “the presence or absence of faces modulates feedforward effective connectivity from the early visual cortex to occipitotemporal areas,” and also determined that information tracts for faces, relative to other objects, are diminished in developmental prosopagnosia. Specifically, they found inferior effective connectivity from the early visual cortex to the bilateral FFA and the right pSTS^9,10^. These authors also found that subjects with developmental prosopagnosia, while having the same overall organization of white-matter as controls in the ventral temporal cortex, displayed similar atypical properties in tracts related to face-selective regions of the cortex^9,10^.

The inferior longitudinal fasciculus (ILF) and inferior frontal–occipital fasciculus (IFOF) are described in previous works^28–30^. Its occipito-temporal course makes it a candidate fiber for the processing of faces. Five different sub-components of the IFOF have been proposed based on diffusion spectrum imaging fiber tracking^30^. Using a combination of tractography and cadaveric dissection, we were able to confirm at least three different sub-components. Components of the IFOF from the lingual gyrus and inferior occipital gyrus were shown to terminate in the inferior frontal gyrus, and components from the cuneus were shown to terminate in the superior frontal gyrus. In terms of the ILF, our tractography and anatomic dissection confirmed the presence of ILF subcomponents. Similar to Latini, we were able to identify a cuneal, lingual, and inferior occipital branch of the ILF^29^. The ILF and IFOF connect many of the aforementioned regions, and studies have found evidence that they are also important for the perception of faces^5^. Individuals with congenital prosopagnosia demonstrate structural disruptions of the bilateral ILF and IFOF, including low fractional anisotropy (FA) and decreased volume of the right ILF as well as decreased volume of the right IFOF^5^.

Despite the highly interconnected nature of this network, there is some evidence that its arrangement is not organized in a strict serial hierarchy, such that face-related information flows from the inferior occipital gyrus (IOG) to subsequent regions. One study, involving resection of the entirety of IOG-faces as well as the posterior 23.3% of the posterior fusiform gyrus-faces, found that those face-specific regions downstream of the resection displayed remarkable resiliency after the operation, indicating the possibility of a non-hierarchical, multiple-route face network^31^. We plan to perform a more detailed analysis of tract volume of the IFOF, ILF, along with their subcomponents, based on these findings.

There are a series of U-shaped fibers between the inferior occipital gyrus and fusiform gyrus that reach the level of the posterior temporal lobe, a finding reported by the work of Catani et al.^32^. There are fibers from the lingual gyrus and inferior occipital gyrus that feed into the anterior fusiform gyrus. The fibers from the inferior occipital gyrus correspond to an area known as LO (lateral occipital), which receives detail, motion, and information from both the dorsal and ventral stream to process the form of objects^26^. The lingual gyrus contains functional areas related to basic visual processing. This gives credence to the theories that facial processing occurs from a feed-forward mechanism from early visual processing areas (LO, V1, V2, etc.) to the fusiform face area^33^. The adjacency of these connections to the posterior temporal lobe also supports the theory that this information feeds into the posterior temporal area. These findings align with the results that Grill Spector et al. reported regarding the white matter connections of the ventral face network^33^. Grill-Spector et al. discovered hierarchal connections from the inferior occipital gyrus and early visual areas. Additionally, their work mentions vertical white matter tracts connecting with this network, which we have confirmed in the vertical occipital fasciculus (VOF). This may allow regions throughout to be part of the attention network to be connected with the facial processing network. Grill-Spector also notes that facial processing may occur through multiple pathways. We have noted that U-shaped fibers that run from early visual processing areas to the anterior fusiform gyrus. The IFOF and ILF have also been implicated in facial processing. Additionally, there are specific tracts that feed from the inferior occipital gyrus and lingual gyrus into the fusiform gyrus. Together, these findings demonstrate that multiple routes for facial processing may exist in the brain.

The face-selective regions of the fusiform gyrus have been implicated in conditions other than prosopagnosia. The FFA volume has been shown to be approximately twice as large in adults diagnosed with Williams Syndrome compared to age-matched controls, despite the fact that the entire fusiform gyrus region is smaller in Williams Syndrome^34^. Williams syndrome is a genetic developmental disorder that affects multiple organ systems. In relevance to this work, people with Williams syndrome have been found to have difficulty performing visual-spatial tasks. Typically, lesions of the parietal lobe affect the ability to perform these tasks. Therefore, this functional deficit in Williams syndrome patients suggests a possible connection between the fusiform gyrus and visual-spatial tasks. Additionally, neurotypical individuals appear to have a more symmetric fusiform gyrus than those with autism spectrum disorder (ASD). A leftward asymmetry of the fusiform gyrus was demonstrated most commonly in ASD subjects, while a minority of ASD subjects with more severe symptoms exhibited atypical rightward asymmetry^35^. The fusiform gyrus’ potential link to Williams syndrome and ASD highlights the role of this region in facial perception and related functions.

### Fusiform gyrus and other sensory functions

The fusiform gyrus is not solely devoted to facial processing. The left occipitotemporal sulcus and the region of the fusiform gyrus lying just medial to it are implicated in lexical processing, specifically in the computation of grapheme description – independent of orientation, location, or font. Reading written words or pseudowords, and the production of written words or pseudowords while spelling is also mediated by these areas^11^. Damage to the left posterior fusiform gyrus result in impairments in the visual processing of high spatial frequencies, including orthographic and non-orthographic processing, which presents as pure alexia in addition to visual agnosia for objects presented at high spatial frequencies^8^. Some individuals, however, have presented with isolated pure alexia after white matter tracts traversing between the left medial BA 37 and right BA 37 were damaged^11^. This indicates the possibility that left medial BA 37 may not be the only area specific to such graphemic description. This finding may be explained by the VOF, which has connections to the posterior fusiform gyrus. Damage to the VOF has been implicated in the production of a pure alexia. We performed tract volume analysis of the VOF, which demonstrates that the right and left VOF have differences in tract volume (Table 2). This wide variance in patients’ tract volume may demonstrate differing functional roles of the VOF for patients. The VOF has been previously described in the literature^36,37^. These studies, based on diffusion-tensor MRI technology, conclude that the VOF connects the fusiform gyrus with unimodal visual association cortices. Our tractography and anatomic dissection confirms this finding by demonstrating connections in the VOF between the fusiform gyrus and the cuneus. Additionally, we demonstrated connections of the VOF within the previously mentioned lateral occipital regions. However, other fibers that course through the fusiform gyrus, such as the ILF and IFOF, may be candidates for lexical processing as well.

**Table 2:** Tract volumes of vertical occipital fasciculus.

Subject	Laterality	Tract volume (cm^3^)
1	R	3,838.12
	L	4,154.30
2	R	5,261.72
	L	4,615.23
3	R	5,295.97
	L	4,593.75
4	R	6,054.68
	L	6,742.18
5	R	4,253.90
	L	3,753.90
6	R	3,335.90
	L	3,378.90
7	R	3,886.72
	L	2,800.78
8	R	5,558.60
	L	5,109.38
9	R	4,202.34
	L	4,402.34
10	R	5,148.44
	L	6,108.38

*R* right, *L* left.

The fusiform gyrus has also been implicated in grapheme-color and tone-color synesthesia, in which the left posterior fusiform gyrus has been found to have increased gray matter volume, while the left anterior fusiform gyrus, as well as the left MT/V5, have decreased gray matter volume^38^. Finally, there is evidence that the fusiform gyrus demonstrates selectivity for human bodies, separate from faces or tools^6^. Studies demonstrate that diminished effective connections in the fusiform gyrus correlated with a body size misjudgment score, which possibly implicates these regions in the development of anorexia nervosa^7^.

The fusiform gyrus is an important region implicated in such tasks as the visual processing of human faces and bodies as well as the perception of stimuli with high spatial frequencies. Our findings contribute to the understanding of the anatomy of this region. A greater comprehension of the structural and functional relationships inherent to this region may support clinical advancements in such areas as surgical planning for glioma resection and functional mapping in awake craniotomies. Furthermore, post-surgical outcomes related to the fusiform gyrus may be better understood in the context of the fiber-bundle anatomy highlighted by this study.
